# Feasibility and acceptability of self-collection of Human Papillomavirus samples for primary cervical cancer screening on the Caribbean Coast of Nicaragua: A mixed-methods study

**DOI:** 10.3389/fonc.2022.1020205

**Published:** 2023-01-20

**Authors:** Emma McKim Mitchell, Katherine M. Hall, Aubrey Doede, Anneda Rong, Michelet McLean Estrada, Orlando Benito Granera, Francisco Maldonado, Hala Al Kallas, Cassandra Bravo-Rodriguez, Mariana Forero, Yolande Pokam Tchuisseu, Rebecca A. Dillingham

**Affiliations:** ^1^ Department of Family, Community & Mental Health Systems, University of Virginia School of Nursing, Charlottesville, VA, United States; ^2^ Department of Family Medicine, University of California San Diego, La Jolla, CA, United States; ^3^ School of Data Science, University of Virginia, Charlottesville, VA, United States; ^4^ Fundación Movicancer, Managua, Nicaragua; ^5^ St. George’s University School of Medicine, Great River, NY, United States; ^6^ School of Arts and Sciences, University of Virginia, Charlottesville, VA, United States; ^7^ Duke-Margolis Center for Health Policy, Washington, DC, United States; ^8^ Department of Infectious Disease, School of Medicine, University of Virginia, Charlottesville, VA, United States

**Keywords:** HPV, cervical cancer, self-collection HPV test, Nicaragua, underscreened

## Abstract

**Background:**

Cervical cancer is the primary cause of cancer death for women in Nicaragua, despite being highly preventable through vaccination against high-risk genotypes of the Human Papillomavirus (hrHPV), screening for hrHPV, and early detection of lesions. Despite technological advances designed to increase access to screening in low resource settings, barriers to increasing population-level screening coverage persist. On the Caribbean Coast of Nicaragua, only 59% of women have received one lifetime screen, compared to 78.6% of eligible women living on the Pacific and in the Interior. In concordance with the WHO’s call for best practices to eliminate cervical cancer, we explored the feasibility and acceptability of self-collection of samples for hrHPV testing on the Caribbean Coast of Nicaragua through a multi-year, bi-national, community-based mixed methods study.

**Methods:**

Between 2016 and 2019, focus groups (n=25), key informant interviews (n=12) [phase I] and an environmental scan [phase II] were conducted on the Caribbean Coast of Nicaragua in partnership and collaboration with long-term research partners at the University of Virginia and community-based organizations. In spring 2020, underscreened women on the Caribbean Coast of Nicaragua were recruited and screened for hrHPV, with the choice of clinician collection or self-collection of samples.

**Results:**

Over the course of the study, providers and potential patients expressed significant *acceptability* of self-collection of samples as a strategy to reduce barriers currently contributing to the low rates of screening (phases I and II). Ultimately 99.16% (n=1,767) of women chose to self-collect samples, demonstrating a high level of acceptability of self-collection in this pilot sample (phase III). Similarly, focus groups, key informant interviews, and the environmental scan (phases I and II) of resources indicated critical considerations for *feasibility* of implementation of both HPV primary screening and subsequently, self-collection of samples. Through phase III, we piloted hrHPV screening (n=1,782), with a 19.25% hrHPV positivity rate.

**Conclusion:**

Self-collection of samples for hrHPV testing demonstrated high acceptability and feasibility. Through concerted effort at the local, regional, and national levels, this project supported capacity building in reporting, monitoring, and surveilling cervical cancer screening across the continuum of cervical cancer control.

## Introduction

Almost entirely preventable through vaccination against high-risk genotypes of the Human Papillomavirus (hrHPV) and through screening and early detection, cervical cancer is a cancer of disparities, with disproportionate mortality in women living in low- and middle-income countries (LMICs). The World Health Organization (WHO) estimates that 85% of global cervical cancer deaths are in LMICs, where women carry a risk of dying from cervical cancer three times higher than that of women in high-income nations (1). In Latin America, cervical cancer is the third most common cause of cancer death for women, but in Nicaragua, it is the leading cause of cancer death for women (2). Within Nicaragua, access to healthcare and preventive services varies geographically, with women living on the rural and remote Caribbean Coast less likely to engage in cervical cancer prevention efforts (3).

Invasive cervical cancer incidence and mortality can be dramatically reduced through early detection and treatment, but many women do not complete screening at recommended intervals (4). Significant decreases in cervical cancer incidence and mortality rates globally are directly attributed to increased screening and early detection (1). The WHO has developed a plan for the elimination of cervical cancer within the next 100 years with specific targets to be reached by 2030, including: reaching 90% of girls by age 15 for vaccination against high risk Human Papillomavirus (hrHPV); 70% of women receiving a high-quality screen for cervical cancer by age 35 and again by age 45; and 90% of women receiving treatment (whether for precancerous or cancerous lesions) (5–7). Researchers have indicated that in some LMICs, a single lifetime screen may be all that is currently feasible (7).

While the HPV vaccine is available in Nicaragua for purchase (8), there is not currently a National HPV vaccination program (9). The cervical cancer control program in Nicaragua therefore centers on organized, opportunistic, population-based screening, and early detection through annual Pap testing/cytology for women ages 25-65, and annual cervical visual inspection with acetic acid (VIA) recommended for women ages 30-50 (9). Intra-country variability in screening coverage is significant. While screening efforts in the Pacific region cover an estimated 34.7% of eligible women within a given year, and where 78.6% of women have been screened in their lifetime, it is notable that screening coverage drops significantly when disaggregating the Caribbean Coast of Nicaragua, where 27.1% of eligible women are estimated to be screened in the span of a year and only 59% of women have had a lifetime screen (9).

In line with the WHO’s call to eliminate cervical cancer, there are innovative technologies and community-based implementation models being trialed globally. Self-collecting samples to screen for hrHPV provides particular promise at mitigating some known barriers to screening engagement found in the literature, where in Latin America specifically embarrassment, privacy concerns, machismo of male partners, and the time or difficulty involved with attending a clinic are well documented in the literature (10). As cost is often a significant barrier for feasible implementation of community-based hrHPV testing, it is important to note that research specifically in Nicaragua, as well as in many other countries, has found community-based hrHPV testing to be a cost-effective approach for cervical cancer control (11).

Perhaps one of the largest benefits, is that shifting community based screening models from Pap/cytology testing or VIA to primary hrHPV screening allows for participants to collect their own sample for testing for the presence of hrHPV, the greatest risk factor in developing pre-cancerous or cancerous lesions (12, 13). hrHPV self-collection is an empirically based strategy shown to increase cervical cancer screening for women in lower resourced settings (4), has been found to have comparable sensitivity and specificity to clinician collection for hrHPV testing (12, 13), and has particular relevance for women who are under- or never-screened (14). In studies conducted with diverse populations, self-collection has shown great promise for improving access to screening for vulnerable populations who live in areas where there is poor access and fewer service providers (12–14). The ability to self-collect samples addresses some individual reasons for not participating in screening at recommended intervals (4, 13). Several European studies found higher screening completion rates in underscreened women who received mailed at-home HPV self-collection kits when compared to mailed reminders to come for in-clinic screening (12–14).

Several studies of women in Latin America have shown promising results regarding acceptability of HPV self-collection from both participant and provider perspectives, citing the increased ease and comfort of self-collection versus clinician-collection (15, 16), as well as the benefit of time, with respect to both travel and personal obligations when kits are delivered through community-based implementation programs (10). The introduction of HPV self-collection has been shown to increase screening coverage, particularly in rural and remote areas (15).

While self-collection of samples have been found to have high levels of acceptability in disparate global settings, variability remains. Nicaragua is an important case-study for examining the acceptability of self-collection. In 2014, Bansil et al. found lower levels of participant acceptability for self-collection of samples for hrHPV testing in Nicaragua when compared to participants in Uganda and India, specifically as acceptability was influenced by fear of pain/discomfort, and by concerns about womens’ ability to collect sufficient samples for testing (17). A 2020 study involving the scaling-up of the same HPV-based primary screening assay/platform (*care*HPV) used in the Bansil, et al., study in Nicaragua recruited and screened 44,635 participants over four years (18). While there was a high level of acceptability for self-collection of samples with participants in Nicaragua (and also with participants in the larger study which included community-based HPV testing in Honduras, El Salvador, and Guatemala), it is important to note that there was no inclusion of women from the Caribbean Coast of Nicaragua in the sample, demonstrating a critical need to explore screening barriers and efforts on the Caribbean Coast (18, 19).

### Caribbean Coast of Nicaragua: Contextual factors impacting acceptability and feasibility

Bluefields is the largest city on the Caribbean Coast of Nicaragua and is the political seat of the Southern Caribbean Coast Autonomous Region (RACCS) which is ethnically, linguistically, and culturally distinct from the rest of the country (20). Mestizo, Creole, Miskitu, Garifuna, Rama, and Mayagna ethnic groups are represented (20). Only within the last three years has there been an overland route connecting Nicaragua’s capital of Managua directly to Bluefields (21, 22).

Barriers to cervical cancer screening, diagnosis, and access to treatment persist in the RACCS, though efforts are underway by the Ministry of Health (MINSA) to increase screening and preventive services (23). Within the city of Bluefields, screening services are provided at no cost through a network of primary care clinics or at the region’s only hospital. For rural surrounding communities, screening services are provided at no cost through MINSA brigades, where healthcare providers travel to these remote areas to provide screening and return for follow-up (23).

In the context of the notable disparity and in-country variability in annual population cervical cancer screening coverage on the Caribbean Coast of Nicaragua, decreased likelihood of a lifetime cervical cancer screen, and findings in other parts of the country that there is not only high levels of participant (18) and provider (17) acceptability, but also cost-effectiveness in implementation (11), further research of this model in the regionally-specific context of the Caribbean Coast is warranted. The purpose of this study was to explore the feasibility and acceptability of community-based hrHPV screening with self-collection of samples among underscreened eligible women and their healthcare providers living on the Caribbean Coast of Nicaragua.

## Materials and methods

We collaborated with several long-term educational and research community-based partners in Bluefields, Nicaragua and the international research team at the University of Virginia (UVA) School of Nursing. Partners instrumental in this study include: the Comisarías de la Mujer, charged with representing and providing services for women and families experiencing intimate partner violence (24); the Bluefields Indian and Caribbean University (BICU) School of Nursing, which is the only school of nursing within the RACCS and has collaborated for over a decade on health and development investigations integrating nursing students from both BICU and UVA (20, 22, 25, 26); and the Centro de Derechos Humanos Ciudadanos y Autonómicos (CEDEHCA), a long-term collaborator with the research team, which supports human rights campaigns with vulnerable populations throughout the Caribbean Coast of Nicaragua. These partnerships reflect a long-term commitment to research capacity building within Bluefields in conjunction with these key governmental and non-governmental organizations.

**Figure 1:**
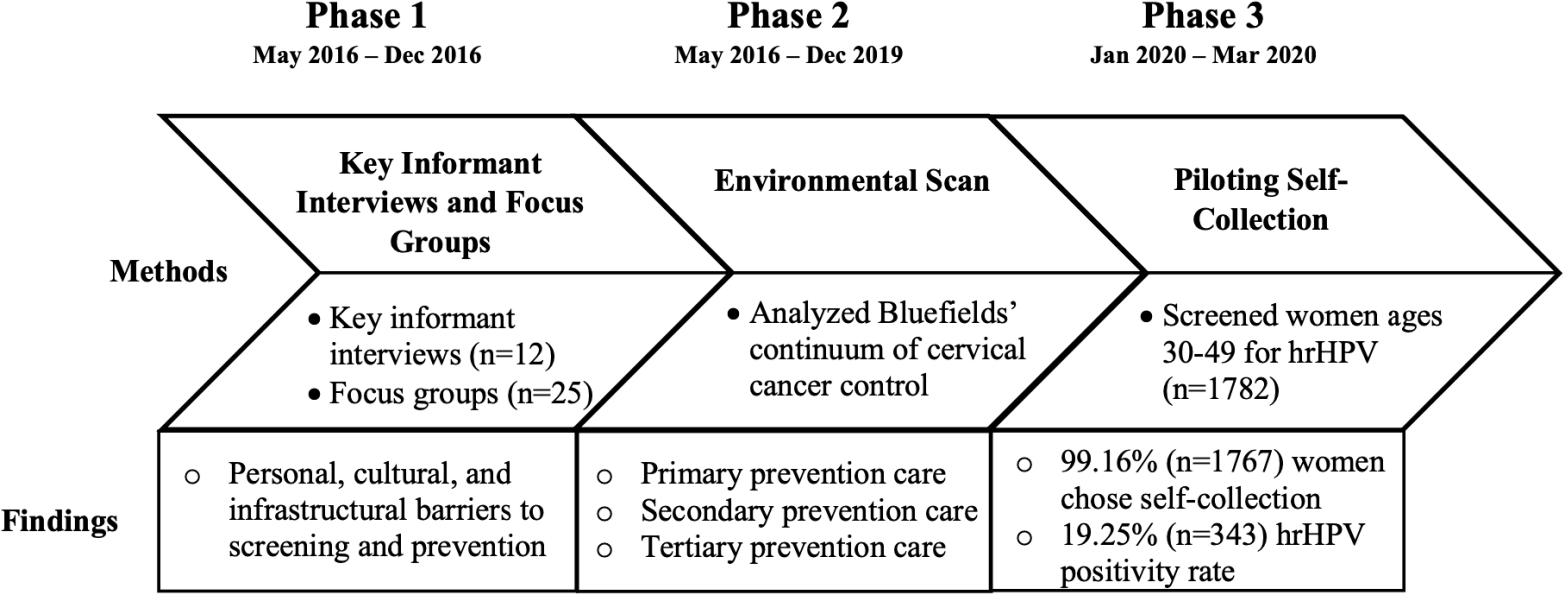
Study phases and timeline.

Beginning in 2016 and continuing to 2022, we conducted a three-phased iterative research study exploring the feasibility and acceptability of self-collection for primary HPV screening in underscreened women on the Caribbean Coast of Nicaragua. Phases I and II involved a mixed-methods, community-based needs assessment conducted through key informant interviews, focus groups, and a systematic environmental scan. In partnership with the Nicaragua Ministry of Health, Phase III involved implementation of HPV primary screening in underscreened women in Bluefields, Nicaragua (please see **Figure 1**). We report herein on specific time points where data were collected, however, it is important to note that this study is embedded within a larger decade-long program of collaborative research and bilateral education initiatives between University partners and community-based organizations and institutions.

### Phase 1: Key informant interviews and focus groups

In the summer of 2016, study team members partnered with the Ministry of Health and conducted a mixed-methods community-based needs assessment through key informant interviews (n=12) and focus groups (n=25 across 5 focus groups). Key informant interviews included Ministry of Health officials, nurses, traditional medicine experts, as well as youth educators. Five focus groups were conducted with cancer survivors, nurses, college students, and women who would be eligible for HPV-based primary screening. To facilitate transparency and fluency, all investigators spoke Spanish and English and a language and cultural interpreter from Bluefields was present during all interviews. The language used in each interview (Spanish or English) was dependent on the preference of the participants. All data were audio-recorded and transcribed verbatim. Using thematic analysis (27), we analyzed the data collected through key-informant interviews and focus groups. Throughout this process, we generated initial codes from our transcripts, and, upon reaching saturation of our data, we identified emerging themes, which are categorized under prevention, screening, and treatment. A sub-analysis was then conducted using the Socio-ecological Model (SEM) (28) to identify barriers to engaging with or accessing screening services at the individual, interpersonal, institutional, and community levels. These findings were used to inform study procedures and considerations for Phase III.

### Phase 2: Environmental scan

From May 2016 through December 2019, we conducted an environmental scan of the continuum of cervical cancer control from awareness of cervical cancer as a public health issue, through engagement with screening and through treatment, guided by the Socio-ecological model (SEM) (28). The SEM framed explorations into barriers and areas for potential intervention at the individual, inter-personal/clinician, institutional, community, and policy levels. From early 2018 through 2019, the environmental scan was also guided by the WHO publication of *Improving data for decision-making: a toolkit for cervical cancer prevention and control programmes* (29). The latter in particular guided our clinician and institutional level indicators, through structured comprehensive collection of data points with the goal of identifying enough contextual practical data for feasible implementation of other models and interventions.

We analyzed clinics in the area to identify the continuum of cervical cancer control through existing preventive services. To address primary prevention for cervical cancer, we systematically assessed all pharmacies in Bluefields to determine how many offered HPV vaccination and what the process was for obtaining it. For secondary prevention, we assessed clinical resources for preventive services, including public and private clinics. We subsequently assessed tertiary prevention to explore follow up procedures through local clinics for follow-up care if a woman were to test positive for hrHPV. Along with data from Phase I, we integrated findings from Phase II to inform development of procedures for Phase III.

### Phase 3: Implementation of HPV self-collection

The UVA research team worked with Managua-based NGO Fundacion Movicancer (Movicancer for short) to explore acceptability and feasibility of implementation of HPV primary screening and self-collection of samples from a procedural and policy perspective, at a regional level in the RACCS and at a National level in the context of the Ministry of Health (MINSA). Between January 2020 and March 2020, we partnered with Movicancer and the MINSA to recruit and screen 1,782 women ages 30-49, who were not pregnant, and were due for cervical screening per existing Nicaraguan National screening guidelines. Women were provided culturally tailored teaching on the procedure for collecting their own sample for HPV primary screening, processed through the *care*HPV platform. Women were then given the choice to have the healthcare provider collect the sample for HPV testing, or to self-collect the sample. Samples were then transported to a laboratory setting and batched for results through the *care*HPV platform. All results were communicated to participants in-person by healthcare providers from the Ministry of Health (MINSA).

## Results

### Phase 1: Key informant interviews and focus groups

Interview participants consisted of key informants (n=12) and focus group participants (n=25 across 5 focus groups). Thematic analysis of interview data indicated several personal, cultural, and infrastructural barriers to cervical cancer prevention and screening (please see **Table 1**). Personal challenges included shame, embarrassment, women’s role as caretakers for others, and past negative experiences with the screening procedure. In terms of healthcare infrastructure, women must overcome institutional-level barriers to access, both physically and financially. Cultural barriers were some of the most significant in gaining access to health care, due to complex issues such as Machismo, cancer and condom use taboos, misconceptions about contraception, and the preference for traditional medicine versus medical-model clinics. In many cases, these barriers are more prominent for women in rural areas.

**Table 1:** Emergent themes and exemplary quotes related to barriers to screening (N=12).

Emergent theme	Exemplary quotes
Personal barriers	
Shame, embarrassment, and shyness	*It’s hard because either they already have kids, and it kind of makes them feel embarrassed because they feel mature to be exposed.*
Negative past experiences	*It is quite difficult, yes, because….they have their experiences with a woman who said that it had been maybe ten years since she had a Pap because when she went to have the Pap, generally in good hospitals … the young male medical students did [the exam] for practice … so when they were using the apparatus to perform the exam, it became caught and unable to be removed, and it perforated. So she was traumatized and never again returned to have the Pap done.*
Role as caretakers in the home, putting family before self	*Because imagine you have a school or a church, and people who are in that area that come to the brigade, and a lot of times it’s mainly men with the children and the boys, a lot of time it’s the sacrifices from the women because she’s pregnant, because she has to take care of the small children.*
Cultural barriers	
Machismo	*Women are not empowered in the rural settings, and it’s incredible, they don’t make any decision at all. They would always come [to their medical appointments] with their man and they are not talking at all—not a word.*
Taboo topics	*You know cancer here, it’s a taboo, people do not like to speak about it, so it’s difficult to know exactly if she has cancer, cervical cancer because people do not like to speak about it … people are more reserved in that sense, sometimes when you hear people pass away, it’s then that you know, ahh she had cancer, but family or friends do not like to speak about it.*
Preference for traditional medicine	*There are people who, yes, especially people from the [rural surrounding] community, when they realize they have this disease, sometimes they do not return because they may have to have chemotherapy. Whatever it is, they don’t return. Rather, they go to their community and begin natural medicines that are safe.* *————————————* *Traditional doctors are from your background [and culture], if you have a headache, they start to ask you so many questions and you end up finding yourself so relaxed and, if you go to a doctor maybe because of the high demand we have here especially in Bluefields, they don’t have much time to take care of you. We have these, if you’ve been to the health center, you have, she’s a doctor, I’m a doctor, and you have another doctor here, the patient sit here and we ask them all the questions, so people don’t really want to answer, because of the fear of my neighbor is listening to what I’m going to answer, so these are some of the points that people refer they go to traditional doctors because of the care, the ethical part, and the confidence.*
Condom use	*I know several people that have told me they use [condoms] only if they do not know if she has something, but after six, five months that they are together, I ask ‘Are you using protection’ and they respond ‘No’.*
Infrastructural barriers	
Long travel times	*There, they are difficult, depending on the road, it will change a bit, because Bluefields has territory, that is 1-day drive, one-day travelling, two day travelling just to get to a certain point, because then you can look at Rama, then the Curba, then Nueva Guinea, then……, then like 8 hours, 4 hours, 5 hours travelling, it’s difficult, and in the rain, the accessibilities is like 0, with water is high, and then with the cars there are problems, to get to them is difficult.* *————————————* *One of the larger problems is the distance. The geographic location between here and the Coast or because here, all transport is by water. It’s nothing like Managua, where you can catch a bus and go to Granada or wherever. Here, no. Here, everything is by boat, and it costs a lot, and it isn’t every day. For example, for the women who [live far away from the river], there is only transportation on Mondays, Wednesdays, and Fridays, and if there is a problem, they have to wait until Friday or until Monday when the boat leaves. So that is an obstacle.* *————————————* *For example, for Corn Island, there it is by airplane, there it is worse because by boat, it’s a travesty, like four to five hours on the ocean, and by airplane, maybe $120 to go and return. The [boats] from the estuary … they cost 600 Córdobas to go, 600 Córdobas to return. If she comes alone or if she comes with someone because she doesn’t want to go alone, they need to come with another person….La Cruz de Río Grande [municipality] as well, I believe the journey is more expensive than the river and much more expensive, than the one from Kukra Hill [municipality]. So while it could be 100 Córdobas to leave and return from the center of town, the women who are more inland [in rural communities surrounding] Kukra Hill who have to come by truck and leave their community and return….generally, that is the challenge with this group of women – those who live more inland are those who have more problems with access.*
Vaccination availability	*There’s a vaccine, it came out in 2006, but there’s no national program in Nicaragua. And it’s available in some pharmacies, but it’s very expensive because you have to get it from Managua, and you have to get it before a woman is sexually active. So by the time the women know that there was a vaccine, usually it’s a bit late to get the vaccine.*
Long wait times for results	*But they have to wait when it come from Managua … so then that’s the reason why it takes two months, two months and a half and then you will call the health center you have to go and ask for the thing.*

From a prevention standpoint, themes related to primary prevention (before exposure occurs), secondary prevention (screening), and tertiary prevention (prevention of further progression of the disease) emerged. For primary prevention, limited access to HPV vaccination (only available for pre-ordered purchase) was compounded by decreased access to comprehensive sexual and reproductive health education in schools or at the community-level. Participants emphasized the need for more health education related to primary prevention of hrHPV and subsequently cervical cancer.

Secondary prevention (screening) and engagement with existing Pap-based cervical screening services, was described in interviews and focus groups as challenging due to individual and clinic-level barriers, including potential for poor previous experiences, perceptions of a lack of confidentiality at clinics, limited clinic staff and hours, and the significant delay in receiving results after screening (estimated by some key informants to be between 30-90 days before results communication to patients with the current model). Impacting engagement with secondary prevention as well as tertiary prevention (preventing further progression of pre-cancerous or cancerous lesions once identified) were both perceived to be significantly more challenging based on the gender of the healthcare provider, from the patient and often also from their partner’s perspective. Further, if a pre-cancerous or cancerous lesions were identified, engagement with tertiary prevention and treatment remained challenging. At the time of data collection, treatment options for anything beyond a pre-cancerous lesion (CIN1) were not available on the Caribbean Coast. For pre-cancerous lesions (CIN1), colposcopy followed by cryotherapy were the recommended treatment pathway. However, with limited trained colposcopists and availability of one colposcope to perform the procedure, as well as delays in accessing gas needed for cryotherapy, this treatment pathway could take a significant amount of time. For more advanced lesions (CIN II/III, ASCUS), women would need to travel to the capital city of Managua for further treatment (it is important to note that during the time span of the phase 2 environmental scan, chemotherapy and later thermal-ablation became available in Bluefields for all women living on the Caribbean Coast). Sub-analyses guided by the Socio-Ecological Model (SEM) are presented in **Table 1** to indicate individual, interpersonal, institutional, and societal level barriers with demonstrative quotes.

In describing challenges and barriers to accessing screening and treatment services in the existing cervical cancer control model, participants clearly indicated the potential role self-collection of samples could play. Self-collection appeared to be a both feasible and culturally acceptable method of HPV testing and a better, more accessible method for screening. Interviewees also provided insight into how self-collection might be best initiated and implemented, with recommendations centered on accessibility. For example, self-sampling kits should be both physically accessible to women through clinics and pharmacies in the area, and the kits must be affordable to the general population. In addition, the process of self-collection must be accessible in terms of clear instructions on how to properly use the kits so that a woman is able to successfully perform the test by herself at home. Finally, women should be able to obtain their results in a timely manner, especially compared to the longer wait times that women currently face using Pap testing.

### Phase 2: Environmental scan

In 2016, study team members analyzed 9 public health clinics, one private health clinic, and two community-based agencies (one targeting comprehensive sexual and reproductive health education, one tracking healthcare service delivery, n=12). Using data collection forms tailored to each type of institution, data were collected on: demographics of catchment areas for each clinic; specialties provided in each (and whether these differed between the public and private clinics); healthcare providers available (nurses, physicians, gender break-downs of each); whether Pap testing/cytology and VIA were both offered for cervical cancer screening; whether colposcopy and follow up treatment were offered at that location; patient costs or fees; and where cervical samples were transferred for processing once collected. Further, at each location study team members explored procedures for identifying underscreened patients, learning that most screening is opportunistic, where patients attend the clinic for another reason, are asked whether they’ve had a Pap test within the last year (verified by chart after self-report), and then offered Pap testing if due. Researchers discussed clinic procedures for a patient who may or may not have been screened previously, and how this data were tracked at a regional level.

For the 10 healthcare clinics in Bluefields targeted, there was a high level of concordance with both regional and National recommendations and guidelines, in terms of initiating screening, screening types (Pap and VIA), recommended screening intervals (yearly), and recommended follow up (colposcopy and cryotherapy when available).

A significant challenge identified consistently was the time interval between sample collection, transportation to the central lab at the regional hospital, and turnaround time for results to be communicated to patients. Adding to phase 1 findings, key informants in phase 2 confirmed wait times of anywhere between 30-90 days before participants knew they did or did not need to follow up in a clinic.

These data served to inform procedures and planning for piloting HPV-based primary cervical cancer screening (phase 3), and in this context a limitation was that there was high variability in healthcare provider availability and subsequently specialties available at each clinic site included in the environmental scan analysis. Further, centralized/Ministry of Health service utilization data were more accurate, particularly when comparing over time, for individual clinic catchment area demographics, than individual clinic assessments reported on here.

In 2018 through 2019, study team members traveled to Bluefields to continue the environmental scan (23), now guided by the WHO’s toolkit on data collection for cervical cancer control (29). All operational public and private clinics were analyzed (n=13) for: transportation considerations (n=13 accessible through taxis, less accessible for rural surrounding communities); potable water (accessible at n=11 clinics); power sources (consistently accessible for n=12 clinics); wifi (n=0 clinics had this accessible) and landline phone access (n=7 clinics). Each of these components is necessary to implement cervical cancer screening and control efforts per the WHO toolkit.

In assessing where cervical screening could take place, and adding this to previous data collection on demographics of catchment areas, the research team partnered with the Ministry of Health (MINSA) to identify target clinics to pilot HPV-based primary cervical cancer screening. Using regional targets for screening coverage, updated clinic catchment-area demographics and priorities, and data points from screening services in 2018 and to that point in 2019, 10 priority clinics in Bluefields were identified as strategic for piloting HPV-based primary screening, including emphasis on the age groups of 30-49 and 50-59 (please see phase 3 below and **Table 2**).

**Table 2:** Characteristics of study participants (*N=1782*).

Sociodemographic characteristics	n (%)
Ages	
< 30	2 (0.11)
30-49	1775 (99.61)
> 50	4 (0.22)
Ethnicities	
Mestizo	1388 (77.89)
Creole	338 (18.97)
Miskitu	46 (2.58)
Rama	6 (0.34)
Garifuna	2 (0.11)
Missing	2 (0.11)
Patient telecommunication method	
Cell phone	1492 (83.73)
Landline	45 (2.53)
None	245 (13.75)
Occupations	
Ama de casa	1269 (71.21)
Other (including merchant, medical professional, technician, administrator, and food business)	513 (28.79)
**HPV self-collection characteristics**	**n (%)**
Collection Method	
Self	1767 (99.16)
Health Personnel	15 (0.84)
Results	
hrHPV Positive	343 (19.25)
hrHPV Negative	1435 (80.53)
Missing	4 (0.22)

### Phase 3: Implementation of HPV self-collection

In partnership with MINSA, SILAIS, and CEDEHCA, over a 5-week period in early 2020, we conducted hrHPV screening with 1,782 eligible women in Bluefields. Of the 19.25% (n=343) of screened women who required follow-up for hrHPV positivity, only 31 didn’t have access to phones and 7 gave landline numbers. The remaining 305 (89%) participants who tested hrHPV positive provided cellular numbers to be reached for follow-up (30). While barriers to accessing existing cervical cancer control screening services persist due to the requirement for clinic-based collection, primary HPV testing for hrHPV allows for self-collection of cervicovaginal samples, previously found to be culturally acceptable in Nicaragua (31). We employed the QIAGEN careHPV ™ assay, and hrHPV positive participants were triaged using VIA and treated, when necessary, with thermoablation (30). We collected study-specific data and utilized the Nicaraguan National Cervical Cancer Surveillance System (SIVIPCAN) to follow patients through the care continuum. We found high provider and patient acceptability of self-collection of samples (99.16% self-collected), but it is important to note that a significant challenge the study team had in monitoring patient follow-up was the coinciding impact of COVID-19 on this region of Nicaragua. Our sample was reflective of the population living in Bluefields and on the Caribbean Coast: 78% identified as Mestiza; 19% identified as Creole; 2.6% identified as Miskitu; 0.3% identified as Rama; and 0.1% identified as Garifuna. Amas de Casa, or women who run their household, were the most represented group in the sample (n=1,269, 71%), indicating this methodology may have particular relevance in targeting groups most at risk for being underscreened, as has been found in prior studies in other locations (10, 13, 16–18).

## Discussion

Women’s access to health services remains a particular challenge to women in rural communities, particularly with reproductive health. The barriers identified in this study are consistent with other studies about cervical cancer screening as well as breast cancer screening (32). Some of these barriers, namely those associated with personal embarrassment, hesitancy to return to a clinic, and machismo, may be reduced or eliminated by providing women with a private and effective method to administer HPV sample collection. In addition, providing a method for self-collection may also address the barriers related to time and personal commitments (travel time to the clinic and the perception that a woman cannot take care of herself because she must care for her family), as this self-sampling may be performed at home and with a significantly reduced time commitment. While some services related to women’s health must still be performed in a clinic, HPV self-collection provides a viable and acceptable method for providing women with an alternative method to screen for a preventable disease. Further, it may even help to connect women to sustained primary care (4).

In Nicaragua, the lack of a national HPV vaccination program leaves primary and secondary screening as the mode of cervical cancer prevention on which most women depend. Novel technology, such as self-collection of cervical samples offer one approach to overcome barriers identified in this study; namely, personal and infrastructural factors that may not allow women to seek timely care. This study describes the particular cultural and geographic barriers to care experienced by women on the Caribbean Coast of Nicaragua and the methods used to integrate HPV self-collection into the country’s existing healthcare system. The data collected here indicate that the majority of women (99.16%) are willing and able to perform self-collection. However, previous studies addressing the acceptability and feasibility of self-collection among Nicaraguan women have shown varying results. For example, while Jeronimo et al. (33) showed an 80% acceptability rate of self-collection, Bansil et al. (17) found that only half of women had a preference for self-collection compared with traditional cervical sampling, with women citing concerns such as an unwillingness to touch the genital region because of shyness or a fear of doing harm. Of note, these studies from Nicaragua do not include the Caribbean Coast, a region with different geographic, cultural, and economic considerations compared with the rest of the country. The current study offers insight into the cultural and practical considerations necessary to implement a public health screening program in this region. With a successful demonstration of the integration of HPV primary screening and self-collection of HPV samples into sustained cervical cancer control, it is possible to have a sustainable program as a result of governmental buy-in of an accepted and validated process.

Successful demonstration projects should rely on geographically relevant input for implementation considerations. Sustainability of integration of new modalities is contingent on governmental buyin and integration, and this will only happen if outcomes and objectives for such programs are collaborative designed and successfully met. In this study, this was done through an iterative and collaborative approach to assessment, analysis, and subsequent procedure design. For example, identifying the need to culturally tailor training materials for participants in instruction on self-collection of samples, the study team sought input from key informants and offered training materials with regionally relevant images, and representative languages (Spanish and Nicaraguan Creole).

### Limitations

One limitation was in the timeline of phase III, necessitated through procurement of supplies necessary to perform molecular testing with the *care*HPV© assay. Manufactured in China, these supplies met manufacturer requirements for implementation for 3 months once they arrived in-country. It is an indication of the expert strategies the nurses and physicians involved in the study utilized that we recruited so many participants in such a short period of time prior to expiration. A significant consideration is in the rapidly changing landscape of cervical cancer control technologies in low recourse settings. For example, our research team began systematically collecting data for the phase II environmental scan before the WHO published the data collection toolkit we ultimately used (23). It is important for cervical cancer control researchers to remain current in implementation of evidence-based prevention strategies.

## Conclusions

It is important to recognize that while populations of women in other regions may have similar experiences regarding their ability to access care, the findings from the current study are specific to the study area, and the proposed self-collection is a product of collaborative development with in-country partners in order to produce an intervention that is both culturally tailored and regionally relevant. Therefore, successful demonstration projects for self-collection in a different region must rely on geographically relevant input and must be approached with cultural considerations specific to that population. Community-based primary HPV screening presents multiple opportunities to mitigate barriers and increase engagement with cervical cancer screening and prevention efforts. Self-collection of samples for HPV testing is not a “one-size-fits-all” or universally acceptable approach. Comprehensive assessments into acceptability, feasibility, and implementation of different community-based cervical cancer prevention efforts are necessary to inform procedures and practices that have a higher likelihood of meeting program goals. Continued research is necessary to guide best-practice in prevention efforts to respond to the WHO’s call to eliminate cervical cancer.

## Data availability statement

The raw data supporting the conclusions of this article will be made available by the authors, without undue reservation.

## Ethics statement

The studies involving human participants were reviewed and approved by University of Virginia SBS IRB. Written informed consent for participation was not required for this study in accordance with the national legislation and the institutional requirements.

## Author contributions

EM and MM made substantial contributions to the conception, design, execution, and dissemination of the work. HAK, CB-R, MF, and YP contributed to data collection. KH, AD, AR, OG, and FM conducted data analysis and interpretation. EM, KH, AD, and RD drafted original manuscript. All authors provided substantial review and revision to the manuscript. All authors contributed to the article and approved the submitted version.
